# The promise of targeting heme and mitochondrial respiration in normalizing tumor microenvironment and potentiating immunotherapy

**DOI:** 10.3389/fonc.2022.1072739

**Published:** 2023-01-04

**Authors:** Zakia Akter, Narges Salamat, Md. Yousuf Ali, Li Zhang

**Affiliations:** Department of Biological Science, The University of Texas at Dallas, Richardson, TX, United States

**Keywords:** tumor micoenvironment, heme, mitochondrial respiration, hypoxia, angiogenesis, cancer immunotherapy

## Abstract

Cancer immunotherapy shows durable treatment responses and therapeutic benefits compared to other cancer treatment modalities, but many cancer patients display primary and acquired resistance to immunotherapeutics. Immunosuppressive tumor microenvironment (TME) is a major barrier to cancer immunotherapy. Notably, cancer cells depend on high mitochondrial bioenergetics accompanied with the supply of heme for their growth, proliferation, progression, and metastasis. This excessive mitochondrial respiration increases tumor cells oxygen consumption, which triggers hypoxia and irregular blood vessels formation in various regions of TME, resulting in an immunosuppressive TME, evasion of anti-tumor immunity, and resistance to immunotherapeutic agents. In this review, we discuss the role of heme, heme catabolism, and mitochondrial respiration on mediating immunosuppressive TME by promoting hypoxia, angiogenesis, and leaky tumor vasculature. Moreover, we discuss the therapeutic prospects of targeting heme and mitochondrial respiration in alleviating tumor hypoxia, normalizing tumor vasculature, and TME to restore anti-tumor immunity and resensitize cancer cells to immunotherapy.

## 1 Introduction

Cancer is one of the leading causes of death worldwide with nearly 2 million new cases in 2022 (1). It is accounted for an approximately 10 million deaths in 2020 (2) and lung cancer alone contributed to 350 deaths per day in 2022 (1). This demands the development of sustainable treatment options to cure and manage cancer. Cancer immunotherapy either in the form of immune checkpoint blockades (ICBs), also known as immune checkpoint inhibitors (ICIs), or chimeric antigen receptor (CAR) T-cell therapy (3) has improved cancer treatment over traditional therapies including chemotherapy, surgery, radiation therapy, and so on. It shows stable clinical responses, progression free survival, and long survival benefits with a greater aim to reactivate the immune system to kill cancer cells (4).

Unfortunately, many cancer patients show resistance to immunotherapies either as primary or acquired resistance. Some patients are unresponsive to cancer immunotherapy treatment at early stages, known as primary resistance, while some patients manifest therapeutic resistance after 8 to 10 months, known as acquired resistance (5, 6). Different types of factors including immunosuppressive TME, high mitochondrial bioenergetics or oxidative metabolism, hypoxia, and tumor vasculature are mechanistically involved in inducing and promoting resistance to immunotherapy (7–9).

Various components of TME such as tumor cells, blood cells, and stromal cells stimulate cancer cells proliferation, migration, and metastasis, affect the infiltration of cytotoxic T cells into cancer cells, and consequently help cancer cells evade the immune surveillance mechanism (3, 10). These cells promote CD8+ T cell exhaustion, immunosuppressive environment, and immunoresistance by releasing proinflammatory cytokines, triggering angiogenesis and hypoxia and up-regulating immune checkpoint proteins (ICPs) such as programmed death-1 (PD-1), programmed death ligand-1 (PD-L1), cytotoxic T-lymphocyte antigen-4 (CTLA-4), lymphocyte activation gene-3 (LAG-3), and T cell immunoglobulin mucin -3 (TIM-3) (11–13). Thus, an interest has grown in the better understanding of the mechanism that modulates TME into immunosuppressive TME.

Cancer cells need high energy for their unregulated cell growth, progression, and metastasis, which cause them to use high mitochondrial bioenergetics and excessive oxygen compared to normal cells (14). Heme plays a central role in mitochondrial respiration by supplying the prosthetic groups of oxidative phosphorylation (OXPHOS) complexes (15). It had been found that cancer cells upregulate mitochondrial respiration, hence uptake more heme compared to normal cells. High ATP demand as well as enhanced oxygen consumption by tumor cells create hypoxia in TME areas. This hypoxic condition makes immunosuppressive TME by upregulating vascular endothelial growth factor (VEGF), resulting in neo-vasculature and activating hypoxia-inducible factor-1 (HIF-1) signaling pathways involved in therapeutic resistance (16, 17).

Hypoxia caused by rapidly proliferating tumor cells triggers the formation of new blood vessels, neo-vascularization, from existing blood vessels into TME, which is functionally and structurally abnormal compared to normal vessels (18). This disorganized tumor vasculature also inhibits the effective delivery of immunotherapeutic agents and other drugs to tumor areas and prevents the infiltration of cytotoxic CD8+ T cells, hence supporting cancer cells survival and progression with limited sensitivity to therapeutics (19).

Therefore, an investigation of the mechanism of an immunosuppressive TME amplified by heme and dysregulated mitochondrial respiration are required to improve the recruitment of effector immune cells into tumor areas, enhance anti-tumor immunity, and resensitize cancer cells to immunotherapy. This review will focus on the role of heme and mitochondrial respiration on mediating immunosuppressive TME and present the promising therapeutic prospects of targeting heme and mitochondrial respiration to normalize TME and potentiate immunotherapy.

## 2 Background of cancer immunotherapy

Immunotherapy has emerged in the last decades as an exciting and promising new modality of cancer treatment and is considered as the “fifth pillar” of cancer therapy. It has a long history, dating back to William Bradley Coley’s use of Streptococcus pyogenes and Serratia marcescens, known as “Coley’s toxins”, which were injected into tumors to treat a variety of malignancies including sarcoma and lymphomas, and this cemented his place as the “father of immunotherapy” (20, 21). However, the modern use of immunotherapies in cancer can be traced back to Dr. James Allison and Dr. Tasuku Honjo’s work on immune checkpoint molecules, culminating in the first ICIs, ipilimumab and nivolumab, and earning the researchers the 2018 Nobel Prize in Medicine or Physiology (22). Immune checkpoint molecules are important proteins that modulate the immune response and allow cancer cells to evade anti-tumor immunity. ICIs are antibodies that target these ICPs to block interaction between ICPs and allow the body to resume an immune response capable of targeting the cancer. The US Food and Drug Administration (FDA) has approved three categories of ICIs such as anti-PD-1 (Nivolumab, Pembrolizumab, and Cemiplimab), anti-PD-L1 (Atezolimumab, Durvalumab and Avelum), and anti-CTLA-4 (Ipilimumab) (4). ICIs targeting CTLA-4, PD-1, and PD-L1 are used for different cancers, including melanomas, breast cancers, and lung cancers (23). The use of ICIs has helped thousands of patients to achieve a stable remission of their cancers.

Other forms of cancer immunotherapy include cytokines that can regulate the host immune response, such as interleukin -2 (IL-2) and interferons (IFNs). IL-2, which was first discovered in 1976, has been approved by FDA for kidney cancer and melanoma (24). IL-2 enhances the effects of T-cell and natural killer (NK) cell activity, allowing the host immune response to target cancers. It is also combined with other immunotherapies, such as ICIs, in order to boost the host immune response and allow targeting of “cold” tumors. Cold tumors are tumors that are poorly responsive to immunotherapy due to an immunosuppressive TME. IFNs work *via* a similar mechanism like interleukins and are also used in cancer immunotherapy (25). Another emerging immunotherapy treatment modality is CAR T-cell therapy enabling the use of the patients' own T-cells which are adapted to target specific cancers. T-cells are isolated from patients’ blood, and then T cells are edited to express a CAR that allows them to bind to specific tumor antigens. These modified CAR T-cells are then infused back into patient (26). There are currently 6 different CAR T-cell therapies approved by FDA to target a variety of blood cancers including acute lymphoblastic leukemia (ALL), non-Hodgkin lymphoma (NHL), and multiple myeloma (26). Cancer vaccines are another form of immunotherapy which can target a host immune response against antigens expressed by different cancers (27). They are generally divided into autologous, made from the patient’s own cells, and allogenic, made from artificial cells. These cancer vaccines hold promise for durable cancer response, including prevention of cancer (28). Immunotherapies have significantly improved the overall survival rate of patients, especially in case of melanoma cancers (29). In non-small cell lung cancer (NSCLC), pembrolizumab increased median progression-free survival to 10.3 months versus 6.0 months in chemotherapy group (30).

Unfortunately, many cancer patients show limited efficacy and adverse effects to immunotherapies due to different factors such as an immunosuppressive TME or mutations limiting the T cell infiltration to tumor cells (31). An adverse effects could be a cytokine storm, a sudden inflammatory response because of activating the immune system that can be dangerous to the host (32). Such adverse events have limited the possibilities of combining different immunotherapies as well as efficacy but significantly increased patients’ health risk. Additionally, not all cancers are responsive to immunotherapies, due to the presence of different immune evasion mechanisms and tumor heterogeneity phenotype in TME that allow TME to be immunosuppressive and resistance to immunotherapeutics. This is an area of major interest for researchers to target TME following combinatorial approaches such as combining immunotherapies with other agents and molecules to turn “cold” tumors into “hot” tumors favoring therapeutic responses to immunotherapy (32).

## 3 The crosstalk between TME and cancer immunotherapy

TME consists of cancer cells, tumor-antagonizing immune cells, such as effector T cells, dendritic cells (DC), NK cells, and tumor-promoting immune suppressive cells such as cancer associated fibroblasts (CAFs), tumor-associated macrophages (TAMs), myeloid-derived suppressor cells (MDSCs), regulatory T cells (Tregs), regulatory B cells (Bregs), and extracellular matrix (ECM) (33, 34). These cells and components of TME work together and their interaction mostly favor the growth of cancer, an immune evasion of cancer cells, and the insensitivity of cancer cells to immunotherapy (35). CAFs stimulate the release of cytokines and growth factors including platelet derived growth factor (PDGF), VEGF, hepatocyte growth factor (HGF), stromal cell-derived factor 1 (SDF1), and so on, which activates endothelial cells, resulting in an enhancement of tumor growth, angiogenesis process, and tumor vasculature. Immunosuppressive cells upregulate immunosuppressive cytokines such as IL-4, IL-10, IL-13 and transforming growth factor beta (TGF-β), stimulate angiogenesis by expressing VEGF and HIF-1α, and express ICPs, which are involved in tumor development, immunosuppressive TME formation, and anti-tumor immunity evasion (11–13, 36). Macrophage stimulates the release of tumor cells in the circulatory and lymphatic system and works as immunosuppressive agents through suppressing anti-tumor response and mechanism (37–39). TAMs are another prominent cancer promoting cells in TME. TAMs are present in two heterogenous form. One is pro-inflammatory M1 macrophage and another one is anti-inflammatory M2 macrophages, which are controlled and regulated by prostaglandin E2 (PE2), TGF-β, IL-4, IL-10, and IL-13, and involved in tumor-promoting activities by secreting anti-inflammatory cytokines such as TGF-β and IL-10 (40). To promote tumor growth and angiogenesis in TME, most of the macrophages in immune system are polarized to M2 macrophage, which suppress anti-tumor immune responses, subsequently releasing tumorigenic factors and promoting the remodulation of ECM, motility of tumor cells, and intravasation (41).

Cytotoxic T-lymphocytes (CTLs), also known as CD8+T cells, and helper T cells, also known as CD4+ T cells (TH1, TH2, and TH17), are the most prominent components of anti-cancer immunity (42, 43). Helper CD4+ T cells and cytotoxic CD8+ T cells mediate anti-tumor immunity through recognizing and killing cancer cells, but immunosuppressive activities within TME makes CTLs dysfunctional as well as causes T cells exhaustion (44, 45). Interaction between PD-1 and PD-L1, mainly expressed by T-cells and cancer cells, respectively, suppresses T-cells and stimulates the survival of cancer cells (46). Cancer immunotherapies mainly works by targeting T-cells and immune cells to restore anti-tumor immunity, promoting T-cells activation and T cell infiltration within the TME (47). Some constituents of TME have the potential to prevent tumor development, hence they are targeted for cancer immunotherapy. These cells in TME prevent tumor generation by releasing cytokine molecules as well as activating cytokines signaling pathways and direct cell contact (48). Many preclinical studies suggested that the inhibition of fibroblasts activating proteins in CAFs stimulate immunosuppression (49–51). Activation of CAFs by inhibiting different signaling pathways including nuclear factor kappa B (NF-kβ), C-X-C chemokine receptor 4 (CXCR4), fibroblast growth factor receptor (FGFR), and Hedgehog using selective inhibitors showed tumor suppressive or antitumor activity (52). CAFs also participate in an immunosuppressive function through suppressing cytotoxic T cell infiltration and function by releasing TGF-β (53). Targeting MDSCs in TME is another potential strategy for cancer immunotherapeutics. Preventing the immunosuppressive activities of MDSCs involves in blockage of MDSCs proliferation, recruitment, and differentiation of MDSCs into mature cells (54). Several studies showed that using inhibitors or neutralizing antibodies against tumor-derived factors like as IL-6, colony stimulating factor 1 (CSF1), and granulocyte-macrophage-colony stimulating factor (GM-CSF) and chemokines such as CXCR2, CXCR4, CC chemokine ligand 2 (CCL2) suppress MDSCs expansion and recruitment (55–59). Many synthetic inhibitors such as COX-2 inhibitors (celecoxib and acetylsalicylic acid), phosphodiesterase-5 inhibitors (tadalafil, sildenafil, and vardenafil) as well as bardoxolone are used to inhibit the immunosuppressive activities of MDSCs, eventually leading to the differentiation of MDSCs into non-suppressive mature cells (60–62).

However, tumor cells in TME remain in hypoxic state due to high oxidative metabolism and comparatively slow growth of blood vessels than the rapid growth of tumor cells. The simultaneous hypoxia and reoxygenation occurring in the TME areas causes the excessive production of reactive oxygen species (ROS) such as superoxide radical (O^2^.-), hydroxyl radical (OH), singlet oxygen (O_2_), and hydrogen peroxide (H_2_O_2_) which induce oxidative stress in the cells of TME that ultimately break both single and double DNA stand, initiate abnormal DNA synthesis, cause genetic instability, and finally generate aggressive tumor phenotype (63, 64). Although ROS plays a significant role in cancer progression, there are numerous studies suggesting that ROS also has negative effect on tumor growth. One study suggested that an excess level of ROS is produced during T cell activation by T cell receptor (TCR) signaling which regulates cell proliferation and clonal expansion and finally prevent progression of cancer and cancerous infection (65–68). ROS is also responsible for the development of cell-mediated and humoral immunity through the activation of B cell and T cell. H_2_O_2_ is a key element of ROS which has a significant role in B cell maturation and activation and enhance B cell receptor signaling (65, 69). ROS also controls tumor growth through creating balance between T cells and modulate T cell apoptosis (70). ROS from TME can spread up the phagocytosis process through recognizing and engulfing cancer cells (71). Moreover, phagocytic cells such as macrophage, monocytes, and neutrophils generate ROS, eventually leading to killing and clearance of damaged cells (72). ROS degrade pathogens through the formation of neutrophils extracellular traps by signaling cascade (73). In TME, excess ROS are also produced during the differentiation of macrophage (M1 and M2) (74, 75).

**Figure 1:**
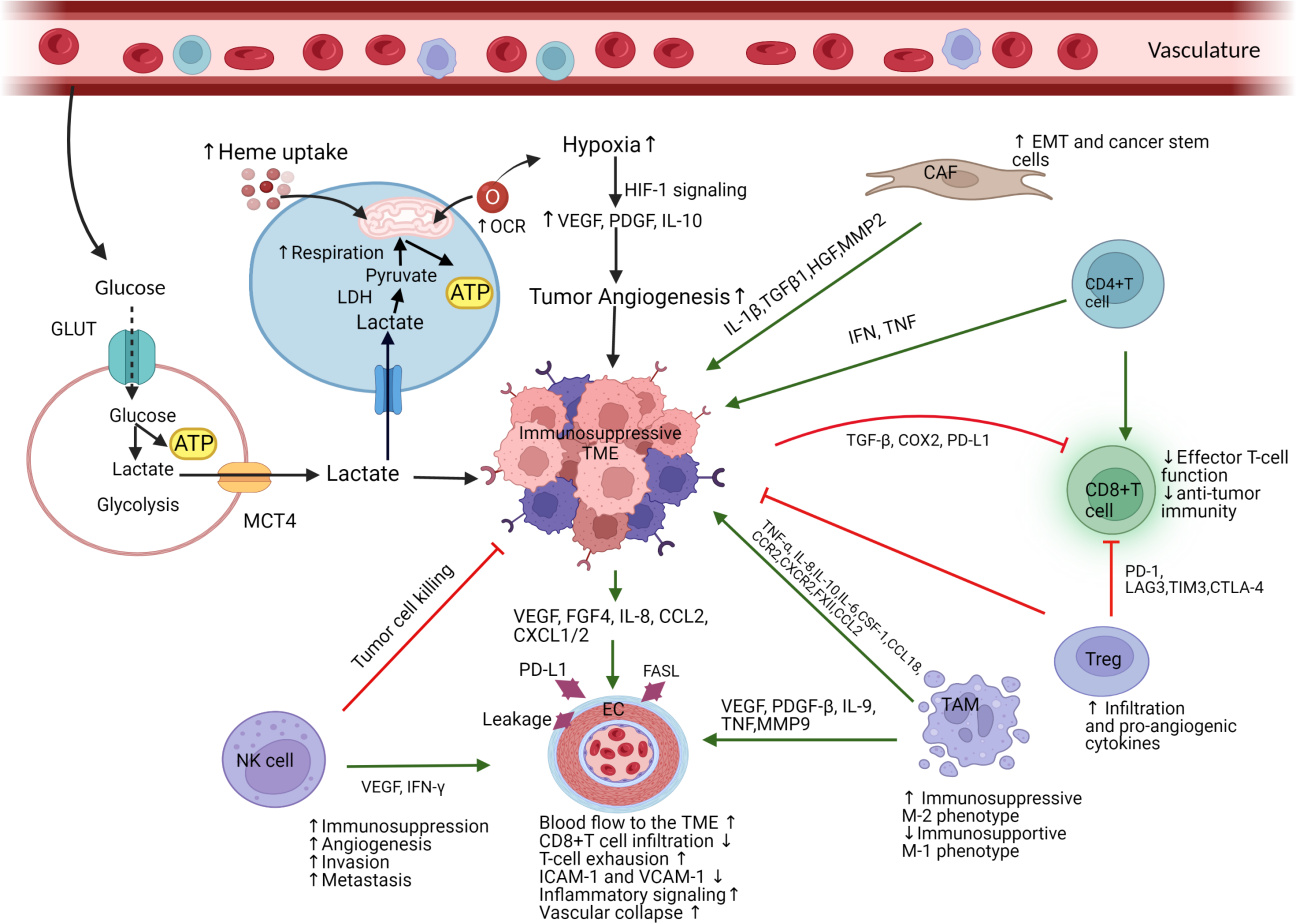
Schematic representation of immunosuppressive TME mainly mediated by tumor cells’ high heme uptake and mitochondrial respiration. (Figure created with BioRender.com accessed on 19 November 2022). Immune suppressive cells and components infiltrate into the TME, interact with tumor cells, causes immune resistance (immunosuppressive), and promote tumorigenesis. Due to excessive consumption of nutrients, tumor cells release lactate which causes acidosis and recruits immunosuppressive cells into TME. Tumor cells’ high heme uptake and mitochondrial respiration associated with an elevated consumption of oxygen promote hypoxia and angiogenesis, which drive an immunosuppressive TME. Hypoxia activates HIF-1 and VEGF signaling cascade which causes dysfunctional vascularization and generates tumor invasion and metastasis. TAMs enhance an immunosuppressive TME function *via* the polarization of immunosupportive M1-like phenotype to immunosuppressive M2-like phenotype. TAMs also promote tumor EC function *via* activating VEGF, PDGF-β, TNF, and IL-9. NK-cells stimulate EC function by targeting VEFG and IFN- γ. Receiving signal from TAMs and NK-cells, tumor endothelial cell become immunosuppressive by increasing blood flow into TME and stimulating the expression of PD-L1 which ultimately increase T cells exhaustion and apoptosis. Immunosuppressive TME causes cytotoxic CD8+ T cells dysfunction and T cells exhaustion. CAFs are responsible for EMT and cancer stem cell generation by activating ECs and releasing cytokines and growth factors which promote tumor growth, angiogenesis, and tumor vasculature. GLUT, glucose transporter; ATP, adenosine triphosphate; MCT4, monocarboxylate transporter 4; LDH, lactate dehydrogenase; OCR, oxygen consumption rate; HIF, hypoxia-inducible factor; VEGF, vascular endothelial growth factor; CAF, cancer associated fibroblast; EMT, epithelial mesenchymal transition; Treg, regulatory T cells; TAM, tumor associated macrophage; EC, endothelial cells; NK, natural killer cells; IL, interleukin; TGF, transforming growth factor; HGF, hepatocyte growth factor; MMP2, matrix metalloproteinase 2; IFN, interferon; TNF, tumor necrosis factor; COX, cyclooxygenase; PD-L1, programmed death-ligand 1; PD-1, programmed death -1; LAG3, lymphocyte activating 3; TIM, T-cell immunoglobulin mucin-3; CTLA, cytotoxic T-lymphocyte associated protein; CSF, colony stimulating factor; CCR, C-C chemokine receptor; CCL, CC chemokine ligand; CXCR, C-X-C chemokine receptor; PDGF, platelet derived growth factor; FGF, fibroblast growth factor; CXCL, C-X-C motif chemokine ligand.

Hypoxia contributes to the generation of immunosuppressive TME and immunotherapeutic resistance by upregulating HIF-1α signaling pathways and triggering the expression of VEGF, PDGF, and other cytokines, resulting in a modification of tumor metabolism, angiogenesis, and lymphangiogenesis (76) (**Figure 1**). Tumor vasculature is structurally and functionally abnormal which leads to the uncontrolled proliferation of endothelial cells and the insufficient oxygen and nutrient supply to immune cells eventually blocking the function of T cells. Moreover, it interferes with the infiltration and delivery of functional T cells towards the tumor, downregulates the expression of intercellular adhesion molecule-1 (ICAM-1) and vascular cell adhesion molecule-1 (VCAM-1), and develops the loss of antitumor immunity and immunotherapeutic resistance (77). Therefore, it is necessary to regulate TME to enhance the sensitivity of cancer cells to immunotherapy by understanding key mechanisms triggering hypoxia, tumor angiogenesis, and other processes mediating immunosuppression.

## 4 Modulation of TME by heme and mitochondrial respiration

Various studies reported that cancer cells import elevated levels of heme and upregulate mitochondrial oxidative metabolism. These cause the modulation of TME into an immunosuppressive state that prevents antitumor immunity and supports cancer cells growth and proliferation, which are discussed into following sections and represented in **Figure 1**.

### 4.1 The immunosuppressive role of heme on TME

Heme is an essential signaling and metabolic molecules that are involved in many biological processes, from metabolism to the regulation of transcription (78) in host. Heme is composed of four pyrrole rings, chelated by a central iron ion. This iron-containing porphyrin has been shown in epidemiological studies to be associated with various diseases such as cancers, Alzheimer’s dementia, vascular diseases, and metabolic diseases (79). Damaged red blood cells release heme, which is known as labile or free heme, into the body that drives immune response and inflammation (80). Labile heme acts as a danger associated molecular pattern (DAMP) that provokes immune responses by binding to toll-like receptor 4 (TLR4) (80). The induction of TLR4 signaling pathways by labile heme causes the release of tumor necrosis factor α (TNF-α) and IL-6 (81), which are associated with immunosuppressive TME and immune evasion (82, 83). Heme also causes endothelial cells dysfunction as well as the induction of endothelial cells or vascular permeability (81). Free heme produces ROS by Fenton reaction, resulting in an induction of oxidative stress and DNA damage (80).

Labile heme also causes the expression of oncogenes and cancer driver genes such as *c-MYC, bcl-2, TGF-β*, and *Wnt* by working on guanine quadruplex (G4) DNA structure as a heme-G4 DNA complex, resulting in a stimulation of cancer cell growth and proliferation (84). It is known that TGF-β promotes immunosuppressive TME and the loss of anti-tumor immunity by triggering the clonal expansion of Treg cells and inhibiting the function of effector T cells and DCs (85). Wnt/β-catenin signaling pathway also influences TME and cancer immunotherapy by inhibiting the infiltration, activation, and proliferation of CD8+ T cells and increasing Treg cells recruitment in TME (86). Therefore, targeting free or labile heme could possibly regulate vascular integrity and the expression of these genes and subsequently these immunosuppressive activities.

Cancer cells display intensified heme uptake and synthesis (79, 87) (**Figure 1**), which is manifested by the overexpression of heme carrier protein 1 (HCP-1) in lung cancer and gastric cancer cells (88). This elevated heme uptake of cancer cells also enhances mitochondrial oxidative metabolism. Heme does not only help coordinate the assembly of several OXPHOS complexes, but it is itself incorporated into complexes II-IV of the electron transport chain (87). This intensified heme uptake and synthesis by cancer cells helps to fuel an elevated ATP production that allows cancer cells to proliferate, and further fuels hallmarks of cancer such as metastasis and angiogenesis (89). Various studies reported the involvement of angiogenesis with immunosuppressive TME and poor immunotherapy responses.

Furthermore, studies in the authors’ lab have shown that heme plays an important role in TME modulation (90). Heme can modulate TME by working on tumor-associated endothelial cells (TECs) and TAMs, that promote angiogenesis and tumor immunosuppression (88) respectively, besides supporting cancer cell growth, survival, and metastasis. There is a metabolic interaction between heme, TECs, and cancer cells (CCs) within TME (91). TECs upregulate angiogenesis through secreting VEGF, PDGF, and FGF molecules and Notch signaling pathways (92). They also promote neovascularization based on CXCR4 signaling pathways and inhibit anti-tumor immunity by preventing cytotoxic reactions of the immune cells (92). Thus, the action of heme on TECs could lead to immunosuppressive activities in TME. Heme causes macrophage polarization, a process that converts macrophages into M1-like and M2-like phenotype macrophage favoring the enhancement of pro-tumor functions and the tumor escape from immune surveillance (88). M2-like macrophages, comprising of TAMs, produce different growth factors, matrix metalloproteinase (MMP), angiogenic factors including VEGF, PDGF, and FGF, and cytokines (93, 94). Taken together, these secreted molecules stimulate tumor cell growth, invasion, metastasis, tissue remodeling, angiogenesis, and immunosuppression within the TME.

Indoleamine 2,3-dioxygenase 1 (IDO1) is upregulated in different types of cancers including lung cancers, ovarian cancers, prostate cancers, colon cancers, breast cancers, and kidney cancers (95). It is a heme-binding enzyme that catalyzes the conversion of tryptophan into kynurenine, an immunosuppressive metabolite. Cancer cells depend on IDO1 for exerting immunosuppressive effects. For instance, IDO1 stimulates Treg cells and prevents the activity and proliferation of effector T cells by producing kynurenine, which leads to an immune evasion and immunosuppression (96). Hence, targeting heme could be another strategy to prevent the binding between heme and IDO1, which may enhance anti-tumor immune responses by preventing kynurenine production and restoring the function of effector T cells.

Heme catabolism also acts as an important factor to modulate TME and immune responses by inducing the expression of heme oxygenase -1 (HO-1) (84). The expression of HO-1 is predominantly triggered by heme which is observed from the immunoblot analysis of tumor lysates collected from a mice treated with heme compared to untreated mice in prostate cancer (84). HO-1 also triggers macrophage polarization, which in turn causes immune suppression in TME areas and immune evasion of cancer cells found in nasopharyngeal carcinoma (97). HO-1 upregulation is associated with oncological events such as tumorigenesis, metastasis, angiogenesis, and hypoxia as well as with poor survival outcome and limited immunotherapy efficacy (98, 99).

HO-1 induction has been shown to be implicated in prostate cancer progression by inducing metastasis (100). HO-1 may also have a direct impact on immune cells. One study found that HO-1 may prevent NK cell function by inhibiting CD48 expression, resulting in an immunosuppression and immune invasion of cancer as shown in both *in vitro* and *in vivo* studies (101). Yet, studies have also shown a dual role of HO-1, demonstrating its mediation of ferroptosis induction, while it may have a protective role in cases of elevated ROS (102). The upregulation of HO-1 showed anti-PD-1 resistance in melanoma and breast cancer mouse models (103), suggesting the role of heme catabolism in limiting immunotherapy response.

### 4.2 The immunosuppressive role of mitochondrial respiration on TME

Dysfunctional mitochondrial metabolism is one of the hallmarks in cancer, leading to an amplification of mitochondrial respiration and many biosynthetic pathways. Alterations of mitochondria, both in the cancer and the immune cells of the TME, leads to an immunosuppressive condition (**Figure 1**) and limited sensitivity to immunotherapies by overexpressing PD-1 and PD-L1 signaling pathways (104). According to various studies, mitochondrial metabolic reprogramming is implicated in triggering resistance to multiple cancer therapeutics, such as radiotherapy, chemotherapy, targeted therapy, and immunotherapy. Song et al., showed that the reprogramming of mitochondria by an epigenetic alternation led to the loss of ATP synthase subunit that secrete ROS and stabilize HIF-1α under hypoxic conditions, perpetuating a hypoxic phenotype (105). This loss of ATP synthase was associated with resistance to several cancer therapies. Therefore, it could be essential to understand the immunosuppressive mechanism of upregulated mitochondrial oxidative metabolism for targeting mitochondrial respiration in order to normalize TME.

#### 4.2.1 Mitochondria modulate T cell response

During cellular stress, mitochondria can be disrupted and release ROS, mitochondrial DNA (mtDNA), and other mitochondrial DAMPs (mtDAMPs) into the extracellular environment (106). These mtDAMPs released mostly in response to severe cellular damage activate an immune signaling pathway that mounts an immune response against the cancer cells (107). In the immediate aftermath of cellular damage and mtDAMPs release, the immune-primed TME leads to a T cell response that allows for targeting cancer cells (108). Unfortunately, a chronic inflammatory state arises from prolonged mtDAMPs signaling that leads to T cells exhaustion and immunosuppressive condition in TME due to the hyperactivation (104). Therefore, targeting mitochondrial mtDAMPs release within a certain threshold to elicit an immune response, yet not cause an immune suppression by chronic mtDAMPs release, can be a therapeutic strategy in targeting “cold” tumors.

#### 4.2.2 High mitochondrial OXPHOS triggers an immunosuppressive TME

Elevated mitochondrial OXPHOS is a hallmark of various cancers, including breast cancer, gastric cancer, hepatocellular carcinoma, and NSCLC (87, 109). Increased oxidative metabolism does not conflict with the fact that most cancer cells exhibit enhanced glycolytic rates, which were observed by Warburg (110). In fact, elevated cancer cells’ glycolysis is linked with an intensified OXPHOS as pyruvate and lactate, products of glycolysis, enter and amplify TCA cycle in nearly all NSCLC tumors (111, 112). Additionally, components of OXPHOS complexes and markers of mitochondrial biogenesis are found to be highly predictive of reduced overall survival rate in NSCLC patients (113).

Studies have shown that elevated tumoral mitochondrial OXPHOS can lead to an enhanced tumor hypoxia (114) associated with an immunosuppressive TME due to the inadequate supply of oxygen to effector T cells. In case of melanoma, tumor oxidative metabolism was found to be associated with limited anti-PD-1 therapeutic responses (115). They demonstrated that the excess consumption of oxygen by tumor cells causes hypoxia and T-cell dysfunction. Interestingly, the suppression of tumor oxidative metabolism decreases hypoxia in tumor areas and consequently improves therapeutic responses to anti-PD-1 treatment (115).

Tumor hypoxia is a common feature among a variety of solid tumors, and many studies have been done aimed at attempting to relieve tumor hypoxia (107) to normalize TME. Hypoxia suppresses the activation of immune cells including helper T cells and cytotoxic T cells, enhances the activity of the immune suppressed cells, and provokes multidrug resistance (MDR) (16). It can not only lead to the expression of T cell inhibitory molecules, but also can cause the recruitment of immunosuppressive cells such as MDSCs, Treg cells, CAFs, and TAMs (114) into TME.

As mentioned earlier, hypoxia induces angiogenesis by stimulating the expression of angiogenic factors (VEGF) (116). Angiogenesis is the creation of new blood vessels surrounding the tumor in order to satisfy its demand for increased oxygen and nutrients. However, the creation of these new blood vessels is not always successful, and in fact, many of these newly formed blood vessels are improperly created which are structurally leaky and impair oxygen delivery to the tumor which in turn mediates increased angiogenesis (104). Angiogenesis leads to immunosuppression by this enhanced cycle of hypoxia signaling and buildup of toxic metabolites in the TME, resulting in an effector T cell exhaustion.

In addition, toxic metabolic waste products from the elevated tumor OXPHOS can exacerbate the immunosuppressive TME. These toxic metabolites can affect T cells, leading to the decreased function of T cells. Further, glucose depletion in the TME, mainly by the energy intensive tumor cells, can lead to the decreased access of glucose to T cells, which make a switch to glycolytic metabolism upon activation and thus have an increased need for readily available glucose. This can decrease T cell activity and lead to the tumor cells to maintain an immunosuppressive TME (114). Enhanced mitochondrial respiration also produces ROS that support cancer cell proliferation, progression, metastasis, and the modulation of various cells residing in TME, leading to immunosuppressive characteristics (117).

## 5 Therapeutic prospects of targeting heme and mitochondrial respiration

Cancer immunotherapy has been showing promising results and durable responses for lung cancer, breast cancer, head and neck cancer, and melanoma in various clinical trials studying the effects of blocking PD-1/PD-L1 interaction (118). However, resistance to immunotherapy is very common phenomenon to majority of cancer patients due to cancer heterogeneity and dynamic TME (35, 119). Different types of cells in TME interact with each other and create a hostile condition to anti-tumor immunity and cancer immunotherapy (35). In addition to making immunosuppressive TME, these mechanisms reduce the supply of oxygen and nutrients to immune cells, prevent the infiltration of effector T cells in tumor areas, and induce immunotherapeutic resistance (11). Therefore, it could be a promising approach to target heme and mitochondrial respiration for overcoming immunosuppressive mechanisms in TME and restoring anti-tumor immunity, which are discussed in the following section and summarized in **Tables 1**, **2**.

**Table 1:** Studies targeting heme and heme catabolism and their effects in TME and anti-tumor immunity.

Therapeutic molecules/Inhibitors	Targets	Experimental models/Cell line	Effects	References
Heme exporter (FLVCR1a) inhibitor	Heme exporter (FLVCR1a)	Endothelial Cells	i. impairs angiogenesis ii. induces metabolic reprogramming	(120)
ZnPPIX	HO-1	Melanoma mice model	i. reduces tumor growth ii. improves anti-tumor immunity iii. increases anti-PD-1 treatment responses	(121)
ZnPPIX	HO-1	Syngeneic murine breast cancer (4T1) model	i. improves CD8+ T cell functions ii. induces the TAMs conversion into M1 macrophages	(122)
ZnPPIX	HO-1	Bone marrow-derived macrophages (BMDMs) isolated from glioblastoma patients	i. reduces macrophage induced immunosuppressive activity ii. inhibits IL-10 expression ii. downregulates STAT3 expression iii. inhibits PD-L1 expression	(123)
Knockout of HO-1 gene expression	HO-1	Lymphoma mouse model	i. increases anti-tumor responses ii. restores the proliferation of cytotoxic T cells iii. reduces immunosuppressive effects in TME iv. increase the therapeutic effects of anti-tumor vaccine OVA	(124)
ZnPP	HO-1	Colorectal cancer cells (HCT-15) HCT-15 xenograft mice models	i. inhibits tumor growth, cancer cells proliferation, and tumor progression ii. reduces hypoxia and angiogenesis through inhibiting HIF-1α and VEGF expression	(125)
ZnPP SnPP Knockout of HO-1 gene expression	HO-1	PDAC cell line, Orthotopic mouse models	i. inhibits PDAC cells proliferation ii. regulates hypoxia iii. increases apoptosis	(126)
CycT	Heme	NSCLC xenograft mouse models	i. inhibits heme synthesis and degradation ii. reduces tumor growth iii. reprogram TME	(127)

**Table 2:** Studies targeting mitochondrial respiration and OXPHOS complexes and their effects in TME and anti-tumor immunity.

Therapeutic molecules/Inhibitors	Targets	Experimental models/Cell Line	Effects	References
HeSP2	Heme OXPHOS complexes	NOD/SCID lung cancer mouse models; NSCLC cell line	i. reduces OCR and ATP levels ii. reduces hypoxia and angiogenesis iii. normalize vasculature	(79, 90)
Mitochondria based tumor vaccine	Tumor-associated mitochondrial Ags (TAMAs)	RENCA cells	i. increases the activity of CD8+ T cell functions ii. improve anti-tumor immunity	(128)
JHU083	Glutamine; Oxidative metabolism	syngeneic mouse models	i. inhibits tumor growth ii. reduce hypoxia iii. restore T-cell metabolism and anti-tumor responses iv. reduces the glycolytic and oxidative metabolism of tumor cells	(129)
IACS-010759	Mitochondrial OXPHOS complexes	Brain cancer and acute myeloid leukemia (AML) model	i. reduces cancer cell proliferation ii. causes apoptosis iii. prevents nucleotide biosynthesis	(130)
CPT	Mitochondrial OXPHOS; Mitochondrial fusion	Orthotopic TNBC mouse models	i.causes autophagy ii. inhibits the conversion of M1 macrophages into M2 iii. enhances anti-tumor immunity	(131)
Metformin	Mitochondrial respiration	Osteosarcoma mouse models	i.reduces immunosuppressive cells, MDSCs, and TAMs ii. lowers OCR iii. inhibits tumor growth iv. improves anti-tumor effects	(132)

### 5.1 Targeting heme and normalizing TME

As mentioned earlier, heme modulates TME, so it might be useful to target heme to normalize TME and improve immunotherapy responses. Multispectral optoacoustic tomography (MSOT) analysis showed the improvement of TME by targeting heme in NSCLC xenograft models (89). Kalainayakan et al. studied the effects of cyclopamine tartrate (CycT), which inhibits heme synthesis and degradation, resulting in a reduction of tumor growth and an improvement of TME areas, in NSCLC xenograft mouse models (127). It had been found that the inhibition of heme exporter, FLVCR1a, in TECs perturbs the metabolic interaction among heme, TECs, and CCs and impairs angiogenesis, resulting in a metabolic rewiring within TME by triggering fatty acid oxidation and ketone bodies accumulation (91, 120).

Various studies reported HO-1 as a potential therapeutic target to improve immunosuppressive TME and restore anti-tumor responses and found promising results both *in vivo* and *in vitro* studies. For instance, TAM-induced HO-1 inhibition or removal of myeloid specific HO-1 led to an enhancement of anti-tumor immunity and anti-PD-1 treatment responses in melanoma cancer model (121).

Kim et al. found that targeting HO-1 using HO-1 inhibitor, zinc protophorphyrin IX (ZnPP), facilitates the conversion of TAMs into M1-like phenotype, resulting in an improvement of cytotoxic T cell functions (122) in syngeneic murine breast cancer (4T1) model. The inhibition of HO-1 prevented immunosuppressive mechanism within TME by inhibiting the expression of IL-10, the upregulation of signal transducer and activator of transcription 3 (STAT3), and expression of PD-L1 in case of gliobastoma found by Magri et al. (123). The downregulation of PD-L1 ultimately could enhance the activity of effector T cells by blocking the interaction between PD-L1 and PD-1.

The pharmacological inhibition of HO-1 from myeloid areas improved anti-tumor responses and anti-tumor efficacy of OVA vaccine by enhancing the proliferation of CD8+ T cells and preventing the immunosuppressive effects of TME (124). ZnPP treatment in colorectal cancer cells and xenograft mouse models inhibits HO-1 that consequently reduces the expression of VEGF and HIF1-α (125). The downregulation of HO-1 by tin protoporphyrin IX (SnPP) was found to control hypoxia and pancreatic ductal adenocarcinoma (PDAC) cell proliferation (126). Bauer et al. showed that the silencing of HO-1 using small interfering RNA (siRNA) causes the inhibition of VEGF driven endothelial cells proliferation by regulating the downstream targets of HO-1, vimentin and calpain (133). As a results, this caused angiogenesis impairment, hypoxia improvement, and tumor growth reduction (125). Taken together, all these studies suggest that targeting heme and HO-1 could overcome the immunosuppressive effects of TME and enhance immunotherapeutic efficacy.

### 5.2 Targeting mitochondrial respiration and normalizing TME

Dysfunctional mitochondrial bioenergetics play a potential role in mediating the loss of anti-tumor immunity and immunotherapy. As stated above, upregulated mitochondrial respiration by cancer cells causes hypoxia within TME areas because of the cancer cells’ elevated oxygen and energy consumption (134). It implies the necessity of increasing the supply of oxygen to immune cells to enhance anti-tumor immunity by targeting the OXPHOS complexes and mitochondrial respiration. Nowadays, the supply of oxygen using nanomaterials are getting attention to mitigate the effects of immunosuppressive TME (135).

Moreover, hypoxia upregulates the expression of VEGF and pro-inflammatory cytokines based on HIF-1α signaling pathways, which orchestrates angiogenesis and the accumulation of immunosuppressive cells within TME (134, 136). These angiogenesis processes represent the loss of anti-tumor immunity and resistance to immunotherapeutics including ICIs and CAR T-cells (134, 136). Different clinical trials are ongoing based on combinatorial approaches following anti-VEGF and ICIs targeting angiogenesis and immune system, respectively (137). However, anti-angiogenics treatment sometimes can prune tumor vessels and other vessels based on dose and time duration, which results in an induction of hypoxia, immunosuppression, and upregulation of PD-L1 (138). Therefore, targeting mitochondrial respiration could be a potential approach to reduce hypoxia and consequently angiogenesis process and immunosuppressive condition within TME areas. It had been found that the use of heme-sequestering peptide (HeSP2) inhibits heme uptake to lung cancer cells, resulting in a decrease of OXPHOS complexes, a reduction of oxygen consumption rate (OCR) and ATP production, and a reduced expression of angiogenic factors (VEGF and VEGFR) (79). Dey et al. showed that the use of HeSP2 reduced angiogenesis, improved hypoxia, and normalized vasculature in NOD/SCID lung cancer mouse models (90). It is worth mentioning that targeting mitochondrial respiration can prevent resistance to antiangiogenics, TKIs (139).

As mentioned earlier, when becoming dysfunctional and damaged, mitochondria release mtDAMP, mtDNA, and so on. Interestingly, one study targeted mitochondrial released abnormal protein, known as tumor-associated mitochondrial Ags (TAMAs), to produce anti-tumor response. Pierini et al. produced a mitochondria based tumor vaccine that showed effective CD8+ T-cell responses and sustainable protection against RENCA cells (128). The inhibition of glutamine using JHU083 lowers the tumor cells oxidative and glycolytic metabolism in syngeneic mouse models, and these effects lead to the reduction of hypoxia, suppression of tumor growth, and the normalization of T- cell metabolism with anti-tumor responses (129).

The use of natural products, cryptotanshinone (CPT), isolated from the herb Salvia miltiorrhiza (Danshen) showed the improvement of anti-tumor immunity through targeting mitochondrial respiration and normalizing TME in orthotopic TNBC mouse models (131). Through targeting mitochondrial respiration, metformin reduced immunosuppressive cells (MDSCs and TAMs) in TME, which results in an improvement of anti-tumor immunity and inhibition of tumor growth (132). Therapeutic benefits of targeting mitochondrial OXPHOS are currently being studied in phase I clinical trials in advanced cancer patients using IACS-010759, an inhibitor of mitochondrial complex I (130, 140).

Interestingly, tumors showed increased sensitivity to therapy when antioxidants were administered that reversed the effects of mitochondria secreted ROS (105). Zhang et al. sensitized MDR breast cancer to chemotherapy doxorubicin by targeting mitochondria using photochemotherapeutic nanoparticles, resulting in an enhancement of anti-tumor immune response and immunogenic cell death (141).

## 6 Future prospects and limitations

Cancer immunotherapy targets the immune system to restore anti-tumor immunity and enable T cells to kill cancer cells; however, immunosuppressive TME limits the effects of cancer immunotherapy and immune responses (142). Hence, combinatorial approaches following the improvement of immunosuppressive TME and anti-tumor immunity could be useful to inhibit the progression of cancer, prevent immunotherapeutic resistance, and ensure sustainable therapeutic benefits with long-term prognosis. As immunotherapy agents mainly work on immune cells, combination of regimens that target different immunosuppressive mechanisms in TME might be useful to enhance the function of effector T cells, prevent the recruitment of immunosuppressive cells, and improve anti-tumor immunity. Many studies are currently ongoing that are aimed at targeting heme and mitochondrial respiration for overcoming therapeutic resistance and improving treatment outcomes. For instance, the use of IDO1 inhibitor is currently under therapeutic intervention as a cancer immunotherapy. This IDO1 inhibitor specifically targets IDO1, which is involved in cancer cells proliferation and immune suppression, and displaces heme from IDO1 by forming a complex (143, 144). It would be possible to improve the efficacy of cancer immunotherapy by regulating heme catabolism because of its role in inducing immunosuppressive characteristics. For example, Schillingmann et al. showed the potentiality of inhibiting HO-1 expression to improve adoptive T-cell responses against cancer patients producing wilms tumor protein -1 (WT1) (145). Synergistic effects of targeting heme catabolism and ICIs were observed in another study. Blockage of HO-1 expression and anti-PD-1 treatment together enhanced anti-tumor effects and anti-PD-1 treatment responses in melanoma mice models (121). However, the dependency of tumor cells and immune cells on oxidative metabolism is very complicated and immune cells need energy for their function, suggesting a judicious strategy for targeting mitochondrial OXPHOS (142). It had been found that the reprogramming of T cells metabolism by targeting mitochondrial OXPHOS increase tumor infiltrating cytotoxic T cells function, anti-tumor immunity, and cancer immunotherapy responses (146, 147). Combination of bezafibrate, which is an agonist of peroxisome proliferator-activated receptor-gamma coactivator 1-alpha/peroxisome proliferator–activated receptors (PGC-1α/PPAR) complexes and selectively targets cytotoxic T cells oxidative metabolism, and anti-PD-L1 amplifies the number of T cells, effector T cells function, and anti-tumor immunity (148). It is also possible to epigenetically target T cells mitochondrial biogenesis, improve effector T cells function, and increase anti-tumor immunity found in an *in vivo* study (149). A number of OXPHOS inhibitors, such as metformin and canagliflozin, are actively being used in clinical trials examining their efficacy in combination with current cancer therapies (16). Notably, metformin combination therapy with anti-PD-1 immunotherapies, nivolumab and pembrolizumab, is being studied in several in-progress phase I and II clinical trials (16). Metformin acts by targeting mitochondrial complex I and thereby reducing OXPHOS, leading to reduced tumor hypoxia, which allows greater T-cell infiltration. Synergistic effects of metformin and IR-780 improved hypoxia by inhibiting mitochondrial respiration and delivering oxygen, respectively. This combination ultimately reduced immunosuppressive MDSC cells, cleared immunosuppressive TME, and enhanced anti-tumor responses (150). Scharping et al. showed that the treatment of metformin with anti-PD-1 reduces oxygen supply to tumor cells and increases the function of T cells (151). Hypoxia-activated prodrug TH-302 was studied in combination with anti-PD-1 or anti-CTLA-4 in prostate cancer mouse model. This combination was shown to reduce 80% tumor, hypoxia and the recruitment of immunosuppressive cells (152). Combinational treatments of IACS-010759, radiotherapy, and anti-PD-1 prevented resistance to anti-PD-1 and improved anti-tumor immunity in NSCLC adenocarcinoma xenograft mice models (153). Therefore, combinational treatments that target heme and mitochondrial OXPHOS along with the immune system could be a novel therapeutic approach by normalizing TME and improving anti-tumor immunity and responses to cancer immunotherapy. It would be desirable to study further in clinical settings to define mechanism and optimal doses, timing, and duration to which targeting heme, heme catabolism, and mitochondrial respiration could potentiate cancer immunotherapy. The heterogeneity of TME and individuals metabolic profiling, which affects therapeutic responses, should be considered before designing any drug regimens for better treatment outcomes and clinical responses. Moreover, some challenges are associated with developing metabolism based therapeutics, for instance, the dependency of both normal and cancer cells on metabolism, the rapid metabolic adaptation of cancer cells, drug delivery issues to mitochondria (154, 155), and so on, which should be addressed before developing anticancer therapies based on metabolic targets.

## Conclusion

Immunosuppressive TME limits the success of cancer immunotherapy. Numerous studies have shown that heme, HO-1, and upregulated mitochondrial respiration play an important role in mediating immunosuppressive TME by promoting the recruitment of immunosuppressive cells, hypoxia, angiogenesis, and immune-evasion in TME areas. Various *in vitro* and *in vivo* studies show that targeting heme and mitochondrial respiration using heme sequestration molecules, HO-1 inhibitor, mitochondrial respiration inhibitor, and inhibitor of OXPHOS complexes could improve hypoxia, normalize vasculature, reduce oxygen consumption of cancer cells, and ultimately restore immunosupportive TME and anti-tumor immunity. However, much more attention is necessary to study the therapeutic effects of targeting heme and mitochondrial respiration and their combination with immunotherapeutic molecules, especially in clinical trials, which can help to develop effective immunotherapeutic drug combinations.

## Author contributions

Conceptualization: ZA and LZ. Literature survey: ZA, N.S, and MA. Writing—original draft preparation: ZA, NS, and MA. Review and editing: ZA, NS, and LZ. Visualization: ZA, NS, and M.Y.A. Supervision: LZ. Funding acquisition: LZ. All authors contributed to the article and approved the submitted version.
